# AI and Machine Learning for Proteomics-Driven Drug Discovery: Methods, Tools, and Best Practices

**DOI:** 10.3390/cimb48050532

**Published:** 2026-05-20

**Authors:** Suman Basak

**Affiliations:** 1 Department of Health Technology (DTU Health Technology), Technical University of Denmark, 2800 Kongens Lyngby, Denmark; sumba@kemi.dtu.dk or basaksuman8@gmail.com; 2 Department of Chemistry, Technical University of Denmark, 2800 Kongens Lyngby, Denmark

**Keywords:** proteomics, mass spectrometry, DIA, drug discovery, machine learning, deep learning, missing data, batch effects, graph neural networks, biomarker discovery

## Abstract

Proteomics has become central to pharmacological research by providing quantitative readouts of protein abundance, post-translational modifications, interactions, and spatial context. However, proteomic datasets are high-dimensional, heterogeneous, and frequently affected by missingness, batch effects, and limited cohort size. Artificial intelligence (AI) and machine learning (ML) can help convert these complex data into decision-relevant outputs for target identification, biomarker discovery, pharmacodynamic monitoring, and drug repurposing. This review critically compares supervised learning, ensemble methods, dimensionality reduction, clustering, deep learning, graph learning, survival modeling, causal inference, and calibration approaches in proteomics-driven drug discovery. We also summarize major software ecosystems for mass-spectrometry processing, targeted assays, spectrum prediction, phosphoproteomics, structure modeling, and reproducible workflows. Emphasis is placed on model selection, benchmarking, missing-data handling, batch correction, interpretability, uncertainty, experimental validation, and translational readiness. Finally, we highlight emerging directions, including contrastive learning, diffusion models, graph-based integration, and federated analytics.

## 1. Introduction

**Figure 1:**
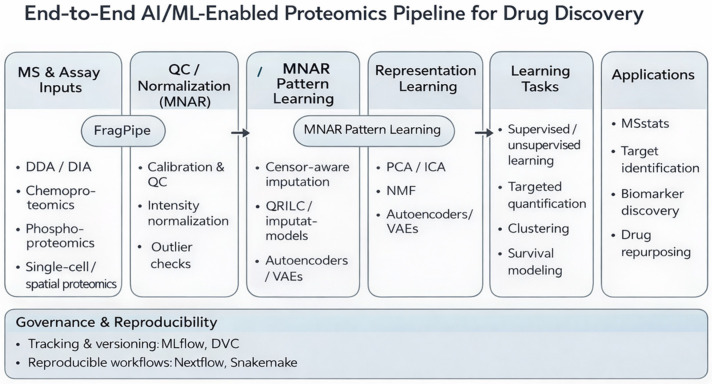
AI/ML-enabled proteomics workflow for drug discovery. Raw mass-spectrometry data from DDA, DIA, chemoproteomics, phosphoproteomics, or thermal-proteome-profiling experiments are processed into peptide, protein, or PTM-site abundance matrices. Representative tools are shown at each analytical layer, including DIA-NN and FragPipe for identification and quantification, Prosit for spectral prediction, DeepPhospho for PTM-focused modeling, Skyline for targeted quantification, MSstats for statistical analysis, and AlphaFold for structure-guided interpretation. The figure highlights where AI/ML outputs enter decision points, including target nomination, biomarker-panel selection, pharmacodynamic monitoring, and compound prioritization for experimental validation. Arrows indicate the progression of the workflow from data generation to downstream applications, while the shaded color-coded modules distinguish major analytical stages. Unlike prior reviews that primarily catalogue AI/ML methods or proteomics tools, this review adopts a decision-oriented perspective. We focus on how proteomics-derived ML outputs can inform concrete drug-discovery decisions, including target prioritization, biomarker panel selection, pharmacodynamic monitoring, and drug repurposing. In addition, we critically compare model families with respect to cohort size, missingness, batch effects, interpretability, calibration, and validation design. This decision-focused framework is intended to help researchers choose methods not only by predictive performance, but also by translational reliability and experimental actionability.

Proteomic experiments, ranging from discovery mass spectrometry (MS), including data-dependent acquisition (DDA) and data-independent acquisition (DIA), to chemoproteomics, phospho-signaling, and thermal proteome profiling, produce matrices of thousands of proteins across hundreds to thousands of samples, often augmented by post-translational modification (PTM)-site resolution and spatial or single-cell context [1,2,3]. The resulting data are high-dimensional (*p* ≫ n), heterogeneous (platforms, labs, batches), sparse with left-censored missingness (limits of detection), and imbalanced (rare events or outcomes) [4]. AI/ML methods address these challenges by (i) learning compact, noise-robust representations, (ii) capturing nonlinear and networked biology, and (iii) delivering calibrated, interpretable predictions suitable for decision-making. The following sections critically compare major AI/ML model families, summarize relevant software ecosystems, and map analytical outputs to concrete pharmaceutical tasks [5]. An end-to-end overview of this AI/ML-enabled proteomics workflow is shown in Figure 1. A practical decision framework linking model families to drug-discovery applications is presented later in this review and can support method selection for new analytical projects.

**Scope and audience.** This review focuses primarily on bottom-up mass-spectrometry proteomics in discovery and translational drug-discovery settings, emphasizing DDA and DIA workflows and their common downstream analysis outputs (protein and PTM-site matrices). We also reference chemoproteomics, phosphoproteomics, and emerging single-cell/spatial proteomics where they motivate specific modeling choices, but we do not aim to comprehensively review all experimental modalities (e.g., top-down proteomics) or every ML architecture. The intended audience includes proteomics analysts, translational bioinformaticians, and drug-discovery teams seeking practical guidance on model selection, evaluation, and deployment readiness.

**Contribution relative to prior reviews.** In addition to summarizing model families and tools, we emphasize decision-relevant best practices—censor-aware missingness handling, batch/shift robust evaluation, leakage prevention, calibration/uncertainty reporting, and reproducible governance—and provide application-algorithm playbooks that map these practices to target discovery, biomarker development, and repurposing.

## 2. Classical Supervised Learning

For a visual guide to method selection across all model families and drug-discovery applications covered in this review, readers are directed to Table 1.

### 2.1. Logistic Regression with Regularization

As illustrated in Figure 1, the methods discussed in this and subsequent sections operate on protein and PTM-site abundance matrices derived after FDR-controlled identification and quantification.

**Overview:** Logistic regression with L1 (lasso), L2 (ridge), or elastic-net penalties remains a reliable baseline for classification (and, with appropriate link functions, regression) on proteomic feature sets. These linear models offer competitive performance while maintaining interpretability and robustness in high-dimensional settings [6,7,8]. In bottom-up MS proteomics, logistic regression is typically applied to protein- or PTM-site abundance matrices derived after false discovery rate (FDR)-controlled identification and rollup. Regularization is particularly important in *p* ≫ n settings common in cohort-scale proteomics, where thousands of quantified proteins are modeled against limited sample sizes. Stability selection can help mitigate feature instability arising from correlated protein modules.

In proteomics datasets, feature matrices often contain correlated proteins derived from shared peptides, pathway co-regulation, and batch-specific signal structure. Consequently, sparsity-inducing penalties such as lasso or elastic-net are particularly useful because they stabilize biomarker selection under correlated feature groups and reduce overfitting in moderate-sized clinical proteomics cohorts.

**Applications:** Typical use cases include building sparse and interpretable biomarker panels for diagnosis and prognosis, predicting treatment response, and quantifying pharmacodynamic readouts.

**Methodological notes:** To mitigate overfitting and support generalizability, employ nested cross-validation for model selection and performance estimation. Report effect sizes and coefficient stability, and consider stability selection to assess feature robustness. For probabilistic outputs, calibrate predicted probabilities using Platt scaling or isotonic regression [9].

Several benchmarking studies suggest that regularized linear models and gradient-boosted trees can be competitive with or outperform more complex models in certain high-dimensional, moderate-sample proteomics settings, though the relative performance varies substantially by cohort, preprocessing strategy, and validation design [6,8]. This observation does not imply universal superiority, but highlights that model complexity does not substitute for careful evaluation design and domain-shift robustness.

Example in proteomics: In large-scale proteomics cohorts such as CPTAC, elastic-net logistic regression models have been widely applied for cancer subtype classification and biomarker discovery, with reported performance varying substantially depending on cohort size, endpoint definition, preprocessing strategy, and validation design. Benchmarking studies indicate that regularized linear models often generalize as well as or better than more complex models in high-dimensional (*p* ≫ n) proteomics settings due to their robustness and reduced overfitting [6,7,8].

When NOT to use: Avoid logistic regression when decision boundaries are strongly nonlinear, when feature interactions dominate, or when sample sizes are very large and nonlinear methods are computationally feasible. Failure modes: coefficient instability under multicollinearity, poor calibration without explicit post hoc correction, and misleading feature rankings when proteins are highly co-regulated.

The sparse protein panels identified by elastic-net logistic regression are directly applicable to the biomarker discovery pipeline described in Section 14.2.

### 2.2. Support Vector Machines (SVMs)

**Overview:** Support vector machines (SVMs) are max-margin learners that employ linear or kernel-based decision functions (e.g., radial basis function [RBF]). They are widely used for classification tasks requiring robustness in high-dimensional spaces [10].

**Applications:** SVMs are particularly effective for medium-sized cohorts with a large number of features, supporting robust classification of disease states and identification of mechanism-of-action signatures.

**Methodological notes:** Linear SVMs are generally preferred when the number of features greatly exceeds the number of samples (*p* ≫ n). Kernel SVMs can capture nonlinear relationships but require careful attention to hyperparameter tuning, feature scaling, and computational costs.

When NOT to use: Avoid kernel SVMs when the number of samples greatly exceeds features, when full probabilistic outputs are required (SVMs do not naturally produce calibrated probabilities), or when interpretability of individual protein contributions is essential. Failure modes: poor generalization if features are not scaled; overfitting with RBF kernels in small cohorts; difficulty in identifying which proteins drive the classification decision.

**Example in proteomics:** In proteomics-based disease classification, support vector machines can be used to distinguish disease and control groups from high-dimensional protein signatures after appropriate feature scaling, missing-value handling, and regularization. In this setting, SVMs support the decision to reduce a broad protein-abundance matrix into a smaller discriminatory protein-signature panel for further biological interpretation or targeted verification. However, because kernel SVMs can be difficult to interpret and tune, linear SVMs are usually preferable when the number of proteins greatly exceeds the number of samples [10].

### 2.3. k-Nearest Neighbors (kNN)

**Overview:** k-Nearest Neighbors (k-NN) is an instance-based learning method that classifies samples based on similarity to labeled training instances [11].

**Applications:** k-NN is commonly used for benchmarking against other classifiers and for subtype assignment after dimensionality reduction or embedding.

**Methodological notes:** The method is sensitive to feature scaling and missing data, and it is rarely suitable as a final model in regulated clinical contexts due to limited interpretability and reproducibility.

When NOT to use: Do not use k-NN as a final model in regulated or translational contexts because it offers very limited interpretability and poor reproducibility. It is also unsuitable when missing data are common, as the method requires complete feature vectors. Failure modes: performance collapses with high-dimensional data if dimensionality reduction is not applied first; results change substantially with small changes in k or the distance metric used.

Comparative positioning of classical supervised methods. The three classical supervised approaches differ in interpretability, cohort size requirements, and handling of correlated features. Logistic regression with regularization is the most interpretable and the most stable in high-dimensional (*p* >> n) settings—it is the recommended first baseline. SVMs offer strong classification margins and work well in medium-sized cohorts, but do not naturally produce calibrated probabilities and are difficult to interpret at the feature level. k-NN is useful only for rapid benchmarking or subtype assignment after dimensionality reduction; it should not be used as a final model in translational settings. In all three cases, missing data must be handled before model fitting, and nested cross-validation is required for unbiased performance estimation.

## 3. Ensemble Methods

### 3.1. Random Forests and ExtraTrees

**Overview:** Random forests are ensemble learners based on bagged decision trees, providing robust predictions across diverse data structures. Extremely randomized trees (ExtraTrees) extend this approach by introducing randomized split selection, enhancing variance reduction [12].

**Applications:** These methods are well suited for modeling nonlinear effects, uncovering interactions, and performing variable-importance screening in support of candidate biomarker and target discovery.

In proteomics-driven drug discovery, tree ensembles are particularly valuable because they can capture nonlinear interactions between protein abundance patterns, post-translational modification (PTM) signals, and clinical covariates such as treatment status or tissue context. This flexibility makes them effective for integrating heterogeneous features derived from mass-spectrometry workflows.

**Methodological notes:** When assessing feature importance, prefer permutation-based measures to mitigate bias introduced by correlated predictors.

When NOT to use: Avoid random forests when full probabilistic calibration is essential without post hoc correction, or when the dataset is very small, as trees may overfit. Failure modes: impurity-based feature importance is biased toward high-cardinality and correlated features—use permutation importance instead; trees may memorize batch-specific patterns if batch variables are not controlled.

**Example in proteomics:** In proteomics-based biomarker discovery, random forests can rank proteins according to their contribution to disease or treatment-response classification. This enables investigators to move from global protein-abundance matrices toward a smaller set of candidate biomarkers for targeted verification. However, because correlated proteins can bias impurity-based importance scores, permutation importance and feature-stability analysis should be preferred when random forests are used for biomarker prioritization [12,13].

### 3.2. Gradient-Boosted Trees (XGBoost, LightGBM, CatBoost)

**Overview:** Gradient boosted trees (GBTs) are sequential ensembles in which each tree is trained to minimize the residuals of the preceding learners. They are among the strongest approaches for structured tabular data [13].

**Applications:** GBTs are widely applied to response and toxicity prediction, as well as to target prioritization tasks that integrate proteomic signals with auxiliary metadata (e.g., tractability, tissue expression).

**Methodological notes:** To prevent overfitting, employ early stopping and use class weights to address class imbalance. Probability calibration is recommended for probabilistic outputs. CatBoost provides efficient handling of categorical covariates such as study site. A compact overview of algorithms versus typical pharma use cases is provided in Table 1.

When NOT to use: Gradient-boosted trees are not appropriate for very small cohorts without strong regularization, or when a fully transparent coefficient-level interpretation is required. Failure modes: overfitting to batch structure if batch variables are not explicitly modeled; poor probability calibration without explicit correction; high sensitivity to class imbalance if class weights are not set.

**Example in proteomics:** In several proteomics benchmarking studies, gradient-boosted tree models such as XGBoost and LightGBM have shown competitive or superior performance in prediction tasks including treatment response and toxicity prediction, though relative gains compared with random forests or linear models depend strongly on dataset size, class balance, preprocessing approach, and validation design [13].

Gradient-boosted models form the backbone of the target prioritization and drug repurposing playbooks described in Section 14.1 and Section 14.3.

## 4. Dimensionality Reduction

### 4.1. PCA/ICA

**Overview:** PCA is an unsupervised dimensionality reduction method that projects data into orthogonal components capturing maximal variance [14,15].

**Applications:** Typical use cases include quality control (e.g., detection of batch drift), coarse structure discovery, and noise suppression in high-dimensional proteomic datasets.

In mass-spectrometry proteomics, PCA is frequently used as a diagnostic tool to identify batch effects associated with acquisition runs, instrument drift, or sample preparation protocols. Visualization of PCA loadings can reveal whether dominant variance components correspond to biological variables or technical artifacts.

**Methodological notes:** Principal components often mix biological and technical sources of variation. Components should be rotated or annotated using available metadata to support correct interpretation.

When NOT to use: PCA scores should not be used as input features in predictive models unless PCA is fitted strictly within the training folds, because otherwise information leakage can occur. Failure modes: dominant components may reflect batch effects or instrument drift rather than biology; loadings become difficult to interpret when proteins are highly correlated.

### 4.2. Non-Negative Matrix Factorization (NMF)

**Overview:** NMF decomposes a data matrix into nonnegative factors, yielding additive component representations that are often biologically interpretable [16].

**Applications:** Typical use cases include discovery of additive transcriptional or proteomic “programs,” such as interferon response or kinase activation signatures [17].

**Methodological notes:** The rank of the factorization should be selected using stability analyses or reconstruction error metrics. NMF tends to align well with pathway-level biology, making it useful for mechanistic interpretation.

When NOT to use: NMF is not appropriate when the data contain negative values (e.g., log-fold-change matrices) or when the goal is supervised classification rather than unsupervised program discovery. Failure modes: results depend on random initialization; the optimal rank is not always clear; components may not be biologically interpretable if missing data are prevalent.

### 4.3. Manifold Learning (t-SNE, UMAP)

**Overview:** Nonlinear embedding approaches such as t-SNE and UMAP reduce high-dimensional data into two- or three-dimensional spaces optimized for visualization of local and global structure [18].

**Applications:** These methods are widely used to visualize subtypes and cellular trajectories, particularly in single-cell or spatial proteomics contexts.

**Figure 2:**
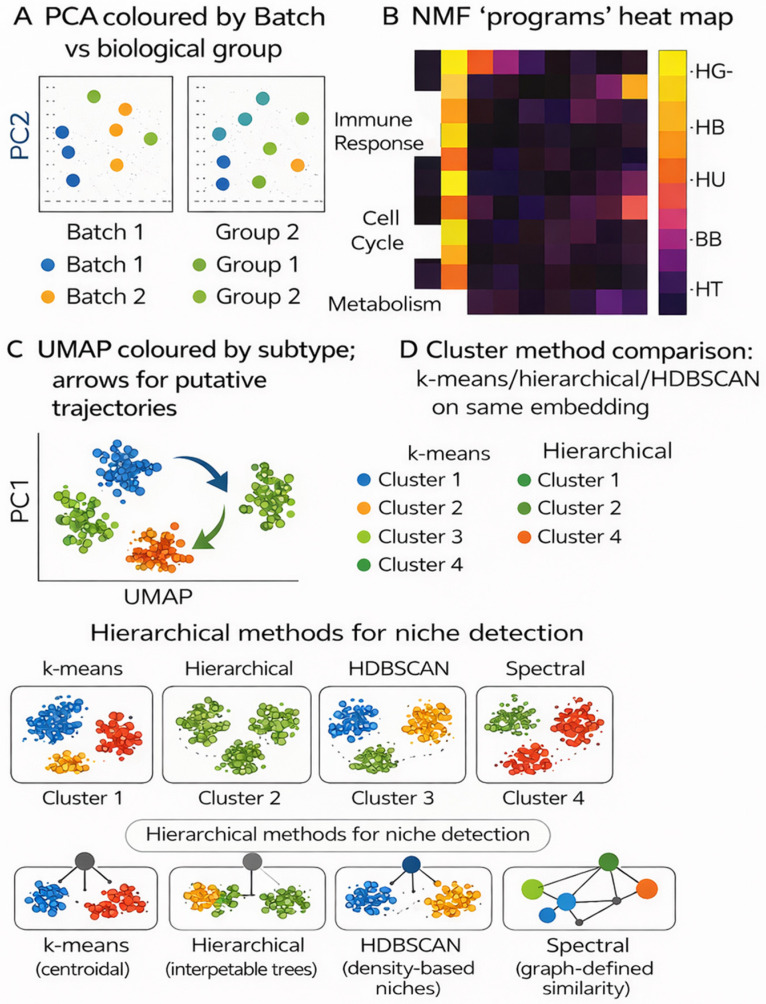
Dimensionality reduction and clustering in proteomics. (**A**) PCA often mixes technical and biological variance; coloring by batch vs. biology reveals drift. (**B**) NMF uncovers additive “programs” aligned with pathways. (**C**) UMAP visualizes subtypes/trajectories. (**D**) Clustering choices shape discoveries: k-means (centroidal), hierarchical (interpretable trees), HDBSCAN (density-based niches), and spectral (graph-defined similarity). Embeddings aid exploration and QC; predictive models should be trained on original features or learned representations, not 2-D embeddings. Colors distinguish the indicated batches, biological groups, clusters, or graph-defined communities within each schematic panel.

**Methodological notes:** Embedded coordinates should not be used as inputs to predictive models, as they are optimized for visualization rather than preserving quantitative relationships. Embeddings are best suited for exploratory analysis. Figure 2 summarizes dimensionality-reduction choices and their implications for clustering.

When NOT to use: t-SNE and UMAP coordinates should generally not be used as input features for downstream predictive models, because the embedding geometry is optimized for visualization rather than preserving quantitative relationships between samples. Failure modes: cluster appearance changes substantially with hyperparameter choices (perplexity in t-SNE, n_neighbors in UMAP); global structure is not reliably preserved in t-SNE; apparent clusters may reflect artefact rather than biology.

The choice between these dimensionality reduction strategies therefore depends on whether the primary goal is quality control and batch assessment (PCA), mechanistic program discovery (NMF), or exploratory subtype visualization (UMAP/t-SNE), as illustrated in Figure 2.

## 5. Clustering

k-means/k-medoids for rapid stratification [19]; hierarchical clustering (Ward linkage) for interpretable heat-maps; Gaussian mixture models for probabilistic membership; DBSCAN/HDBSCAN for irregular, density-based niches [20,21]; and spectral clustering when similarity is graph-defined (e.g., correlations or PPIs) [22]). Applications include disease subtype discovery, co-regulated modules, and microenvironment identification in imaging MS [23,24,25].

When NOT to use: Clustering should not be used when a supervised label already defines the groups of interest, as clustering may not recover those groups reliably. Avoid hierarchical clustering with very large datasets due to computational cost. Failure modes: k-means and k-medoids require the number of clusters to be specified in advance and are sensitive to initialization; all methods are sensitive to missing data and feature scaling; clusters discovered in discovery data frequently do not replicate in independent cohorts without pre-specified validation.

## 6. Deep Learning

### 6.1. Convolutional Neural Networks (CNNs)

The deep learning architectures described in this section represent the model families shown in the middle layers of Figure 1, operating on raw spectral data, image data, sequence context, and protein abundance matrices.

Despite promising results in spectrum prediction and imaging proteomics, deep architectures remain sensitive to instrument-specific domain shift, labeling chemistry, and acquisition regime. In many translational cohorts, limited sample size constrains their effective deployment, necessitating transfer learning or strong regularization.

Unlike many biomedical datasets, proteomics data arise from complex signal-processing pipelines that include peptide identification, protein inference, and intensity normalization. Deep learning models must therefore account for hierarchical structure in the data (spectra → peptides → proteins), as well as instrument-specific variability that can limit cross-laboratory generalization.

**Spectral learning:** 1D CNNs learn from raw/binned MS/MS spectra for peptide identification and intensity prediction [26].

**Figure 3:**
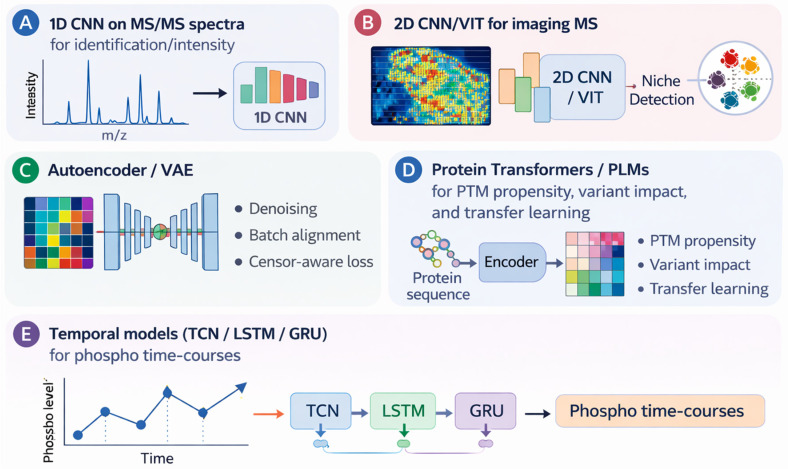
Deep architectures tailored to proteomics. (**A**) 1D CNNs model MS/MS spectra. (**B**) CNN/transformers parse imaging proteomics. (**C**) Autoencoders/VAEs support denoising, batch alignment, and left-censor-aware objectives. (**D**) Protein language models transfer sequence priors to small proteomic cohorts (PTM/variant tasks). (**E**) Temporal models (TCN/LSTM/GRU) capture pharmacodynamic trajectories. The figure highlights that different deep architectures are appropriate for different data layers; CNNs are most relevant for spectra and images, whereas protein language models and GNNs are more relevant for sequence- and network-informed target assessment.

**Imaging proteomics:** 2D CNNs and vision transformers parse imaging MS and multiplexed immunoassays to detect tissue niches. Deep architectures commonly used in proteomics are schematized in Figure 3.

**Example in proteomics:** Deep learning approaches have demonstrated substantial improvements in spectrum prediction and peptide identification. However, in downstream tabular proteomics tasks, deep learning models often do not consistently outperform simpler methods under strict validation conditions, highlighting the importance of dataset size and domain shift considerations [26].

When NOT to use: 1D CNNs for spectral tasks require large, consistently acquired training datasets and are not appropriate when instrument types, fragmentation methods, or acquisition settings differ substantially between training and deployment. For downstream tabular protein-abundance tasks, CNNs offer no clear advantage over simpler models. Failure modes: strong instrument-specific domain shift; overfitting to acquisition artefacts; poor generalization across laboratories without transfer learning or domain adaptation.

Together, these architectures illustrate that deep learning is most powerful in proteomics when matched to the appropriate data layer—spectral prediction and imaging for CNNs, sequence and PTM tasks for protein language models, and network-based inference for GNNs—as depicted in Figure 3.

### 6.2. Temporal Models (LSTM/GRU, Temporal Convolutional Networks)

**Overview:** Temporal convolutional networks (TCNs) are deep learning architectures that leverage dilated causal convolutions to model sequential dependencies in time-series data [27].

**Applications:** TCNs are particularly suited to time-course phosphoproteomics and longitudinal pharmacodynamic studies, where dynamic patterns over time are critical.

**Methodological notes:** Compared with recurrent neural networks (RNNs), TCNs often train more efficiently and generalize well, making them attractive for large-scale temporal modeling.

When NOT to use: Temporal models require time-course data with sufficient time points and replicate structure. They are not appropriate for single-timepoint cohort proteomics. Failure modes: data requirements are high; small time-course datasets lead to overfitting; models are sensitive to irregular sampling intervals and missing timepoints.

### 6.3. Autoencoders and Variational Autoencoders (VAEs)

**Overview:** Autoencoders and variational autoencoders (VAEs) are neural network architectures that learn compressed latent representations through encoder–decoder frameworks [28].

**Applications:** In proteomics, autoencoders are used for denoising, batch effect alignment, and imputing left-censored values when trained with censor-aware loss functions.

**Methodological notes:** To promote meaningful representations, latent dimensions should remain compact and models should be regularized to prevent memorization of the training data.

When NOT to use: Autoencoders are not appropriate as a substitute for principled missing-data imputation when the missingness mechanism is well characterized and simpler methods are available. Failure modes: without regularization, autoencoders memorize training data; VAE latent spaces may not align with biological variables; censor-aware training requires careful loss function design.

### 6.4. Transformers and Protein Language Models

**Overview:** Transformer architectures rely on self-attention mechanisms and have been trained at scale on massive sequence corpora, enabling powerful contextual representations [29,30].

**Applications:** In proteomics, transformer-based models support tasks such as post-translational modification (PTM) propensity prediction, protein function annotation, variant impact assessment, and transfer learning to improve performance on small cohorts [31].

**Methodological notes:** Pretrained models can be fine-tuned on domain-specific data, but careful regularization and benchmarking against simpler baselines are essential to avoid overfitting and to ensure interpretability.

When NOT to use: Transformer-based protein language models are not appropriate for direct prediction from protein abundance tables—they operate on sequence inputs. Fine-tuning on very small proteomic cohorts risks overfitting and should always be compared against simpler baselines. Failure modes: overfitting on small fine-tuning datasets; computational cost; limited interpretability of attention weights as biological evidence.

### 6.5. Graph Neural Networks (GCN, GraphSAGE, GAT, R-GCN)

**Overview:** Graph neural networks (GNNs; e.g., GCN, GraphSAGE, GAT, R-GCN) leverage message passing to model relationships across structured biological networks, including protein–protein interactions, kinase–substrate graphs, and drug–target–disease knowledge graphs [32].

**Applications:** GNNs support tasks such as target ranking, pathway activity estimation, and link prediction for drug repurposing or novel association discovery.

**Figure 4:**
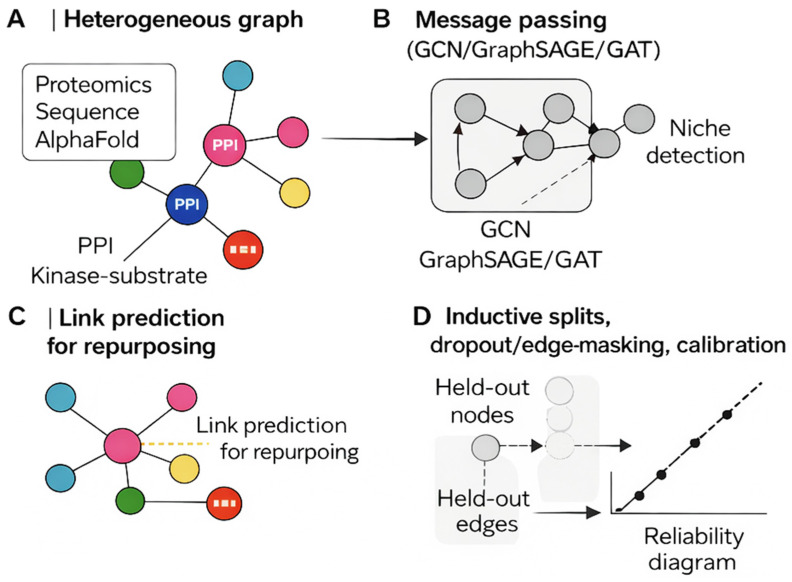
Graph learning on biological networks. (**A**) Heterogeneous biological graph construction; (**B**) graph neural-network message passing; (**C**) link prediction for repurposing; and (**D**) inductive evaluation with held-out nodes/edges and calibration. Proteomic, sequence, and structure-derived features decorate nodes/edges within PPIs and drug–target–disease graphs. GNNs propagate context to rank targets and predict new links for repurposing. Robust evaluation uses inductive splits with held-out nodes/edges; uncertainty is communicated via calibrated probabilities. Arrows indicate information flow or predicted relationships, circles represent network nodes, and different colors are used to distinguish node or feature categories schematically.

**Methodological notes:** To ensure robust generalization, adopt inductive evaluation strategies (e.g., holding out nodes or edges). Apply dropout and edge masking during training to mitigate overfitting and improve model robustness. We illustrate graph learning on biological networks in Figure 4.

When NOT to use: GNNs are not appropriate when the underlying biological network is of poor quality, highly incomplete, or dominated by literature bias toward well-studied proteins. They are also not suitable as a replacement for simpler models when the question does not involve relational or topological reasoning. Failure modes: high-degree nodes (well-studied proteins) dominate message passing; evaluation using random edge splits inflates performance—inductive node/edge holdouts are required; results are sensitive to network source and edge weighting.

The graph-learning approaches described here are revisited in the context of drug repurposing in Section 14.3 and in the network tools discussion in Section 13.6.

Figure 4 illustrates why inductive node/edge holdouts and calibrated uncertainty reporting are essential requirements for reliable graph-based inference in proteomics, and why network quality directly limits the conclusions that can be drawn.

### 6.6. Generative Models (GANs, Diffusion)

Generative modeling in proteomics is a rapidly developing but still emerging area; the following provides a focused overview of the most relevant approaches rather than a comprehensive treatment, as systematic validation in drug discovery contexts remains limited.

**Overview:** Generative models such as generative adversarial networks (GANs) and diffusion models learn to approximate the underlying data distribution, enabling the creation of realistic synthetic samples [33].

**Applications:** In proteomics, generative approaches can be applied to augment rare classes, or to simulate hypothetical perturbations for example, modeling proteome “reversal” under targeted interventions.

**Methodological notes:** Model training requires careful monitoring to avoid mode collapse (GANs) or excessive sampling costs (diffusion). Generated data should be validated against held out biological signals and used to complement, not replace, empirical measurements.

As discussed in detail in Section 18 and Section 23, deep learning does not consistently outperform regularized linear models or gradient-boosted trees in moderate-sized tabular proteomics cohorts. Deep learning is most justified when large training resources, raw spectra, image data, sequence context, network structure, or transfer learning opportunities are available. For protein-abundance matrices with limited sample size, simpler models should remain mandatory baselines.

When NOT to use: Generative models should not be used as a primary data source—they complement but do not replace empirical measurement. This is an emerging area in proteomics with limited systematic validation in drug discovery settings; apply with caution. Failure modes: GANs are prone to mode collapse (generated samples lack diversity); diffusion models are computationally expensive; generated data may introduce artefacts that create false associations in downstream analysis.

## 7. Survival, Calibration, and Causality

**Survival modeling:** Elastic-net Cox proportional hazards models provide transparent baselines for time-to-event analysis. More flexible approaches such as random survival forests and gradient-boosted survival models capture nonlinearities and interactions. Neural network extensions, including DeepSurv and DeepHit, generalize hazard modeling to deep architectures [34,35].

**Calibration and uncertainty:** For probabilistic outputs, calibration is essential. Isotonic regression or Platt scaling provide straightforward adjustments, while conformal prediction, Bayesian neural networks, Monte Carlo dropout, and deep ensembles offer case-level confidence estimates. Such approaches are critical for clinical adoption, where well-quantified uncertainty underpins decision making.

**Causal inference:** To address confounding and distinguish correlates from plausible drivers, causal frameworks are required. Methods include propensity score weighting, causal forests, double/debiased machine learning, Bayesian networks, and target-trial emulation strategies. These tools support the design of analyses that move beyond association toward causal explanation.

In proteomics, confounding is a common and underappreciated problem. For example, in a drug-treated cohort, disease severity may correlate with both treatment allocation and protein abundance, making it impossible to determine whether a protein changes because of the drug or because of underlying disease progression. Standard ML models cannot distinguish between these possibilities—they learn associations, not causes. Causal frameworks address this directly. Propensity score weighting re-weights the sample so that treated and untreated groups are balanced on observed confounders, enabling fairer comparison. Causal forests extend this by estimating heterogeneous treatment effects—identifying which patient subgroups show the strongest protein response to a given intervention. Double/debiased machine learning further allows flexible ML models to be used for nuisance estimation while retaining valid statistical inference for the causal parameter of interest. In drug discovery, these methods help prioritize proteins that are more likely to be mechanistic drivers of disease rather than passive correlates, directly improving the quality of target nominations.

## 8. Handling Missingness, Batch Effects, and Confounding

**Left-censoring (MNAR):** Missing not at random (MNAR) values, such as those arising from limits of detection, can be addressed using quantile-regression imputation, hurdle/two-part models, or censor-aware architectures such as variational autoencoders (VAEs) with censored-loss functions [36].

**Batch effects:** Technical variation across runs or laboratories is frequently corrected using empirical Bayes approaches such as ComBat, removal of unwanted variation (RUV), or adversarial autoencoders that align batches while aiming to preserve biological signals.

**Figure 5:**
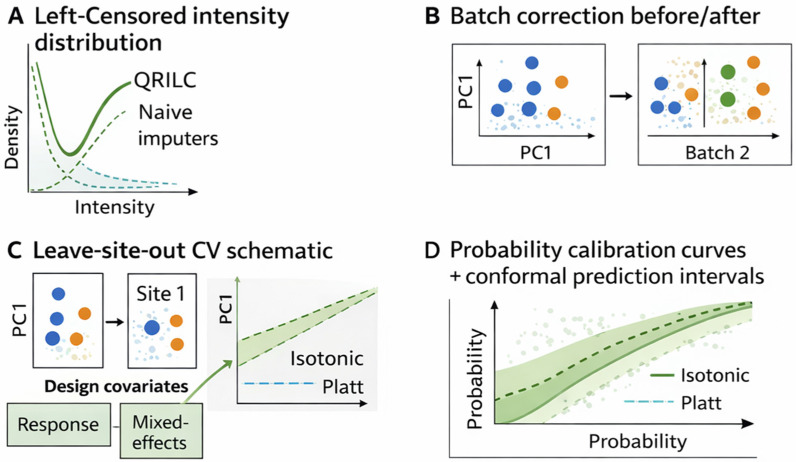
Practical controls for real-world proteomics. (**A**) Left-censoring (MNAR) requires censor-aware methods (e.g., QRILC, hurdle losses). (**B**) Batch correction reduces non-biological drift while retaining signal. (**C**) Confounding is mitigated by design covariates, mixed-effects models, and leave-site-out validation. (**D**) Calibrated and uncertainty-aware predictions (isotonic/Platt; conformal intervals) are essential for clinical readiness. Arrows indicate analytical transitions or model workflows, different colors distinguish schematic groups or methods, and dashed lines denote comparative or reference trends.

**Confounding:** To reduce bias, include design covariates such as study site, age, and sex in models. Mixed-effects formulations can explicitly account for hierarchical structure, while leave-site-out validation helps assess generalizability across cohorts [37]. Practical controls for these issues are summarized in Figure 5.

**Diagnosing missingness mechanisms.** In practice, missingness often mixes missing at random (MAR) and missing not at random (MNAR) processes. A useful diagnostic is to examine missingness probability versus observed intensity (or precursor signal) and to test whether missingness correlates with batch/run/site or phenotype. Strong intensity-dependence suggests left-censoring (MNAR) and motivates censor-aware approaches (e.g., QRILC, two-part/hurdle models, or censored-loss architectures). More sporadic missingness with weak intensity dependence is more consistent with MAR and may justify multiple imputation or low-rank approaches.

**Sensitivity analysis.** Because imputation can induce artificial separability—especially when missingness correlates with batch or outcome—we recommend sensitivity analyses in which key conclusions are compared across at least two defensible strategies (e.g., censor-aware vs. conservative/no-imputation with models robust to sparsity).

The missingness and batch correction strategies described here are embedded as mandatory preprocessing steps in all three application playbooks in Section 14.

## 9. Multi-Omics Integration

**Correlation-based:** Canonical correlation analysis (CCA) and its kernelized variants link proteomic profiles to transcriptomic or metabolomic measurements, highlighting coordinated changes across molecular layers [38].

**Latent-factor:** Methods such as MOFA/MOFA+ and iCluster/DIABLO learn shared low-dimensional representations across omics, enabling identification of cross-modal programs and stratifications [39].

**Graph-based:** Heterogeneous knowledge graphs can integrate proteins, genes, compounds, phenotypes, and side effects. When paired with graph neural networks (GNNs), they provide a structured framework for predicting novel associations and mechanisms.

**Optimal transport:** Optimal transport frameworks quantify how perturbations, such as drug treatments, “move” disease proteomes toward healthy states, offering an interpretable geometry for therapeutic evaluation.

Although multi-omics integration is conceptually attractive, practical implementation in drug discovery is often limited by cohort alignment, missing modality coverage, and batch heterogeneity across omics layers. Consequently, reported gains may reflect confounding rather than true cross-modal synergy.

## 10. Model Explanation and Governance

**Explainability:** Interpretable machine learning is essential in translational proteomics. Techniques such as SHAP values, permutation importance, integrated gradients, and concept activation vectors can map model predictions to proteins, pathways, or cell types.

**Pathway attribution:** Feature importance scores can be subjected to pathway enrichment analysis, providing a mechanistic grounding that links model decisions to biological processes.

**Governance:** Robust governance ensures reproducibility and accountability. Recommended practices include versioning of data and code (e.g., DVC, MLflow), locked preprocessing pipelines, containerized execution environments (Docker or Conda), and workflow engines such as Nextflow or Snakemake to support auditable and transparent analyses.

**Proteomics-specific caution.** Because proteomic features are often highly correlated (co-regulated proteins, shared peptides, pathway modules), per-feature attributions can be unstable and may shift across correlated feature groups. We recommend reporting explanation stability (e.g., rank correlation of top features across folds/resamples, stability selection for sparse models, and pathway-level aggregation) rather than presenting a single attribution plot as definitive.

## 11. Hyperparameter Optimization and AutoML

**Optimization:** Efficient hyperparameter search is essential for performance tuning. Bayesian optimization frameworks (e.g., Optuna, Hyperopt) provide sample-efficient exploration, while bandit-based approaches such as Hyperband or ASHA enable scalable early stopping and resource allocation [40].

**AutoML:** Automated machine learning (AutoML) systems are valuable for rapid baseline construction, provided strict nesting of model selection and evaluation is enforced. To ensure clinical and translational relevance, AutoML pipelines should incorporate domain-specific constraints—for example, encouraging parsimony in biomarker panels—and be paired with robust cross-validation.

## 12. Benchmarking, Datasets, and Evaluation Standards

Fair comparison of AI/ML methods in proteomics depends on evaluation schemes that reflect clinical and pharmaceutical deployment. Random train–test splits often inflate performance because samples from the same batch, site, or acquisition run share technical signatures. Prefer cohort-level, site-level, or time-split validation where feasible, and report performance variability across folds and external datasets.

Evaluation should extend beyond discrimination metrics such as AUC to include precision–recall AUC for imbalanced outcomes, calibration (reliability curves, Brier score), and decision-analytic measures (e.g., decision-curve analysis) when models are intended to guide downstream experiments or clinical actions. For biomarker panels, stability of selected features across resampling, assay feasibility, and robustness to missingness and batch effects should be reported. When proteomic identification uncertainty is relevant, explicitly describe peptide/protein FDR control and how quantification uncertainty propagates into the ML stage.

Public benchmark resources such as CPTAC tumor proteomics datasets and ProteomeTools synthetic peptide libraries have enabled systematic comparison of spectrum prediction models (e.g., Prosit) and DIA scoring tools (e.g., DIA-NN). Reported improvements include increased peptide identification rates and improved FDR control under strict target-decoy evaluation. However, cross-lab generalization remains variable, highlighting the importance of domain-shift-aware validation.

Transparent reporting standards are increasingly recognized as essential for reliable machine-learning applications in proteomics. Recent studies emphasize clear documentation of dataset partitioning strategies (e.g., random splits versus batch, site, or time-based holdouts), preprocessing procedures applied within training folds (normalization, batch correction, imputation, and scaling), and the nesting of feature selection and hyperparameter tuning within cross-validation.

Robust studies further report uncertainty estimates on performance metrics—such as confidence intervals derived from repeated or nested resampling—and validate findings under domain-shift or external cohort evaluation. Adoption of these practices improves reproducibility and enables meaningful comparison between algorithms across proteomics studies.

A task-oriented comparison of major AI/ML model families, including their strengths, weaknesses, validation requirements, and drug-discovery decision relevance, is provided in Table 1 and discussed further in Section 23.

## 13. Tools and Platforms

The tools and platforms described in this section correspond to the processing and analytical layers shown in Figure 1, from raw mass-spectrometry data through to decision-relevant model outputs.

### 13.1. MS Data Processing and Quantification

Widely used tools for mass-spectrometry data processing include MaxQuant/Andromeda for data-dependent acquisition (DDA) identification and quantification [41], DIA-NN for neural scoring and library-free DIA analysis at cohort scale [42], and FragPipe (MSFragger + Philosopher) for rapid database searches, open modification analysis, and PTM characterization via PTM-Shepherd [43]. Commercial packages such as Spectronaut (DIA) and Proteome Discoverer, as well as open frameworks including OpenMS and EncyclopeDIA/OpenSWATH, support modular or library-based workflows. Key tools and platforms by task are summarized in Table 2.

### 13.2. Targeted Assays and Statistics

Skyline provides a comprehensive environment for selected reaction monitoring (SRM), parallel reaction monitoring (PRM), and data-independent acquisition (DIA) assay design and analysis [44]. Statistical toolkits include MSstats/MSstatsPTM for differential and PTM-aware analyses, and Perseus for downstream statistical exploration of MaxQuant outputs [45].

### 13.3. Spectrum/Peptide Property Prediction

Deep learning predictors such as Prosit estimate fragment ion intensities and retention times [46], improving peptide identification confidence and false discovery rate (FDR) control. Related spectral and retention-time models extend this capability across diverse peptide chemistries [47].

### 13.4. PTM and Phosphoproteomics

Deep learning platforms like DeepPhospho predict phosphopeptide spectra and behaviors, supporting kinase–substrate inference. Motif and context-based models include NetworKIN/KinomeXplorer, MusiteDeep, and NetPhorest, while PTM-Shepherd enables large-scale PTM characterization. Curated resources (e.g., phospho-focused databases) further aid biological interpretation.

### 13.5. Protein Structure and Biophysics

AlphaFold delivers high-accuracy protein structures, enabling mapping of variants and PTMs in structural context. Structural descriptors (e.g., pocket detection via fpocket or ML-based predictors) inform druggability assessment and downstream machine learning, such as gradient-boosted models for ligandability ranking [48].

### 13.6. Networks and Knowledge Graphs

Tools such as Cytoscape v3.10.4 enable interactive network visualization, supported by resources such as STRING v12.0, Reactome v96, and BioGRID v5.0.257. Enterprise graph stores, including Neo4j v2026.04.0 and TigerGraph Server v4.1.4 LTS, can be paired with graph neural networks (GNNs) for drug repurposing and target discovery [31,49,50].

### 13.7. Single-Cell and Spatial Proteomics

Pipelines for carrier-based single-cell MS (e.g., plexDIA, SCoPE-style workflows) and imaging MS toolkits facilitate analysis at single-cell or spatial resolution. Dimensionality reduction and clustering implemented with UMAP, using umap-learn v0.5.12, and HDBSCAN, using hdbscan v0.8.43, combined with deep learning support niche detection and tissue context mapping [51].

### 13.8. Workflow and MLOps

Reproducible and auditable pipelines are enabled by workflow engines such as Nextflow v26.04.1 and Snakemake v9.21.0, containerized and environment-management platforms including Docker Engine v29.5.1 and Conda v26.5.0, and experiment tracking/data versioning platforms such as MLflow v3.12.0, Weights & Biases Python SDK wandb v0.27.0, and DVC v3.67.0.

### 13.9. From ML Output to Drug-Discovery Decision

In drug discovery, ML predictions are useful only when they change a downstream decision. Therefore, proteomics-derived models should be evaluated according to the decision they support. For target identification, the output is not merely a ranked protein list, but a prioritized set of targets for orthogonal validation by chemoproteomics, CETSA, TPP, CRISPR perturbation, or functional assays. For biomarker discovery, the ML output should guide selection of a compact assay-ready panel suitable for SRM, PRM, or DIA validation. For drug repurposing, model outputs should prioritize compounds for experimental testing based on disease-signature reversal, target engagement evidence, pathway restoration, and safety constraints. This decision-oriented framing helps distinguish statistically significant proteomic associations from actionable drug-discovery hypotheses.

Practical examples of decision impact: In proteomics-driven drug discovery, ML-supported outputs can influence several concrete decisions. For target identification, proteomic differential-expression, chemoproteomic target-engagement, or thermal stability profiles can be integrated with network propagation or supervised ranking models to prioritize proteins for perturbation, CETSA, thermal proteome profiling, or functional rescue experiments. For biomarker discovery, sparse linear models or gradient-boosted trees can reduce thousands of proteins or PTM sites to compact panels that can be transferred to targeted selected reaction monitoring (SRM), parallel reaction monitoring (PRM), or data-independent acquisition (DIA) assays. For drug repurposing, disease-associated proteomic or phosphoproteomic signatures can be compared with compound-perturbation signatures to prioritize drugs that reverse disease states or restore pathway activity. These examples illustrate that the value of ML lies not only in prediction accuracy, but also in whether the output changes target nomination, assay development, compound prioritization, or validation strategy.

Published examples illustrate this decision pathway. In ML-guided proteomics workflows, classification models can therefore help prioritize disease-associated protein signatures for targeted SRM, PRM, or DIA validation, while repurposing analyses can guide compound selection for experimental follow-up. These examples illustrate that the value of ML lies not only in prediction accuracy, but also in whether the output changes target nomination, assay development, compound prioritization, or validation strategy.

## 14. Application–Algorithm Playbooks

**Figure 6:**
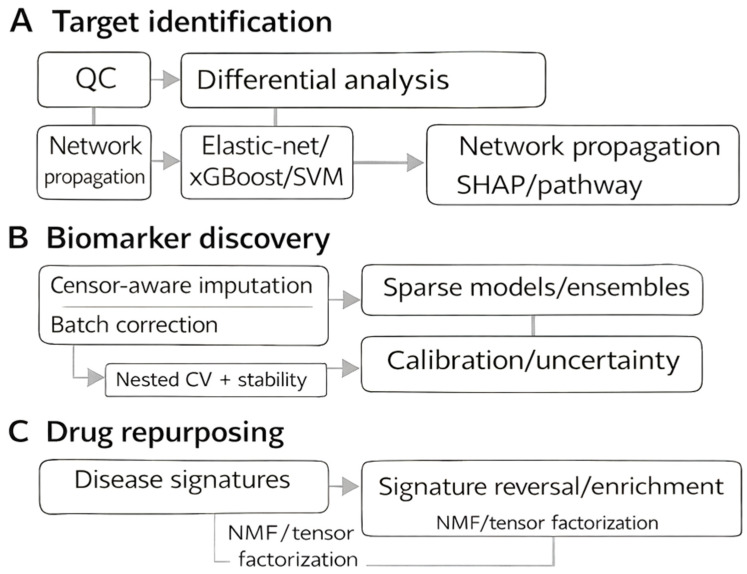
Algorithm-to-application playbooks. (**A**) Target identification integrates differential analysis, network propagation, and supervised/GNN models with interpretable attributions and orthogonal validation. (**B**) Biomarker discovery emphasizes parsimony, nested CV, calibration, and translation to targeted assays. (**C**) Drug repurposing matches disease proteomic signatures to compound effects using correlation/enrichment, factorization, knowledge-graph learning, and optimal transport, followed by triage with ADMET/tissue constraints. The figure links algorithmic outputs to experimentally testable actions, emphasizing that ML-generated rankings require orthogonal validation before drug-discovery decisions are made.

Each playbook below follows a consistent logic: the choice of ML model (Section 2, Section 3, Section 4, Section 5, Section 6 and Section 7) is determined by data modality and cohort characteristics, as summarized in Table 1; model outputs are linked to a specific drug-discovery decision; and each decision maps to an experimental validation strategy described in Section 15. This chain—model → output → decision → validation—is illustrated in Figure 6, which should be consulted alongside each playbook.

Practical model selection guide—choose based on your data and goal: (1) Small cohort, interpretability required → elastic-net or sparse linear model. (2) Moderate cohort, nonlinear effects expected → gradient-boosted trees with nested cross-validation. (3) Raw MS/MS spectra or imaging data available → CNN or transformer-based deep learning. (4) Biological network available, goal is target ranking → GNN with inductive node/edge evaluation. (5) Time-course or longitudinal data → TCN, LSTM/GRU, or survival models. (6) Goal is to identify causal protein drivers, not just correlates → causal forests or double machine learning.

### 14.1. Target Identification and Validation

**Objective:** The goal is to prioritize proteins by therapeutic potential, accounting for mechanism of action, tractability, and safety.

**Pipeline:** A representative workflow begins with quality control and normalization, followed by differential analysis (e.g., limma). Signals are then contextualized through network propagation methods such as random walks or diffusion kernels. Supervised learning models (e.g., elastic-net, XGBoost, SVM) can incorporate tractability and tissue-specific covariates. Graph learning approaches (e.g., GCN, GAT) are applied to protein–protein interaction or kinase–substrate networks, with node features derived from proteomics, sequence data, and AlphaFold-based structural descriptors. Model interpretability (e.g., SHAP values, pathway attribution) supports mechanistic insight, and candidates are validated experimentally using chemoproteomics, CETSA, or thermal proteome profiling (TPP) [52,53,54,55]. Figure 6 links algorithms to these applications.

**Algorithms:** Common algorithms include elastic-net regression, SVMs (linear or RBF kernels), random forests, gradient boosting machines, NMF and autoencoders for program discovery, graph neural networks for topological inference, and causal forests or double machine learning to address confounding.

**Uncertainty propagation into ML.** Proteomics measurements carry uncertainty from peptide-spectrum matching and FDR control, protein inference/rollup, and quantification (e.g., interference, missingness, and normalization choices). Downstream ML should therefore avoid treating protein intensities as error-free. Where feasible, incorporate replicate structure, peptide-level information, or uncertainty-aware summaries (e.g., weighting features by identification/quantification confidence or performing sensitivity analyses across plausible preprocessing choices).

### 14.2. Biomarker Discovery

**Objective:** The goal is to derive compact, robust biomarker panels for diagnosis, prognosis, or pharmacodynamic monitoring.

**Pipeline:** A representative workflow begins with censor-aware imputation and batch correction, followed by feature selection using filter or wrapper approaches (e.g., mRMR, Boruta, recursive feature elimination). Candidate features are modeled with sparse linear methods (lasso, elastic-net, sPLS-DA) or tree ensembles. Nested cross-validation and bootstrap resampling are applied to assess stability. Final models undergo probability calibration (isotonic regression or Platt scaling) and uncertainty quantification (e.g., conformal prediction). Translation to targeted SRM, PRM, or DIA assays can be performed with Skyline, with statistical validation using MSstats [56].

**Algorithms:** Core methods include lasso/elastic-net regression, sparse PLS-DA, random forests and ExtraTrees, gradient boosting frameworks (XGBoost, LightGBM, CatBoost), survival models (Cox regression, DeepSurv), and UMAP/HDBSCAN for subtype exploration.

### 14.3. Drug Repurposing

**Objective:** The aim is to identify novel indications for existing drugs by aligning disease-associated proteomic signatures with compound perturbation profiles.

**Pipeline:** Starting with disease signatures (differential proteins/PTMs, pathway scores), candidate compounds are identified through signature reversal strategies such as rank-based anti-correlation and enrichment. Matrix and tensor factorization (e.g., NMF, CP/Tucker) reveal latent drug–disease axes. Knowledge-graph methods (TransE, RotatE, ComplEx, or GNNs) extend these relationships, while optimal transport quantifies the “reversal distance” between disease and perturbed states. Final prioritization incorporates ADMET properties and tissue-expression profiles, modeled with gradient-boosted rankers. Experimental validation may include restoration of phospho-signatures or direct target engagement assays [57,58].

**Algorithms:** Key methods include correlation and enrichment analyses, NMF/tensor factorization, knowledge-graph embeddings and GNNs, optimal transport, and gradient-boosted ranking models.

## 15. Experimental Validation of ML-Derived Proteomics Hypotheses

The validation approaches described here represent the downstream experimental actions that each ML output in Section 14.1, Section 14.2 and Section 14.3 is designed to inform. Computational predictions in proteomics-driven drug discovery require orthogonal experimental validation before they can support therapeutic decisions. For target identification, candidate proteins should be validated using perturbation assays, CRISPR/RNAi knockdown, chemoproteomics, CETSA, TPP, affinity enrichment, or functional rescue experiments. For biomarker discovery, selected protein panels should be transferred to targeted SRM, PRM, or DIA assays and evaluated for analytical validity, including limit of detection, reproducibility, linearity, and inter-batch precision. For drug repurposing, predicted compound effects should be tested by measuring restoration of disease-associated proteomic or phosphoproteomic signatures, direct target engagement, and phenotypic rescue in relevant cellular or animal models. Thus, ML should be viewed as a prioritization layer rather than a replacement for experimental validation.

## 16. Validation and Common Pitfalls

Robust validation requires strict nesting of model selection within cross-validation, ideally complemented by external cohort replication. Failure to isolate preprocessing steps within folds can lead to optimistic bias, particularly in high-dimensional proteomic settings.

**Multiple testing and FDR:** Control peptide- and protein-level false discovery rates separately. Report effect sizes and uncertainty, not just significance.

**Leakage:** Lock preprocessing pipelines to prevent test data leakage. Avoid using test sets for imputation, scaling, or parameter estimation.

**Confounding:** Include relevant covariates and consider propensity weighting. Assess model transportability with leave-site-out cross-validation.

**Interpretability:** Attribution methods can be unstable; corroborate results with perturbation-based tests and orthogonal assays [59].

**Clinical readiness:** Ensure documentation of analytical validity (LoD/LoQ, linearity, precision), clinical validity (AUC, calibration), and clinical utility (decision-curve analysis).

## 17. Discussion: Practical Tradeoffs for Translational Proteomics

Despite rapid progress, deploying AI/ML in proteomics for drug discovery requires careful tradeoffs between predictive performance, interpretability, and robustness under real-world variability. For many pharmaceutical decisions, strong baselines remain competitive once strict nested validation and cohort-level holdouts are applied—as discussed in detail in Section 18 and Section 23. Deep learning can be advantageous when large labeled datasets exist (e.g., spectrum prediction, image-based proteomics, sequence-centric transfer learning), but it is more sensitive to domain shift across instruments, protocols, and laboratories.

The decision framework in Figure 6 summarizes these practical tradeoffs and provides a visual guide for method selection across the three primary drug-discovery applications. A recurring challenge is that proteomic features are often correlated, missingness is frequently MNAR (left-censored), and preprocessing choices can dominate downstream model behavior.

Accordingly, the most reliable pipelines lock preprocessing, prevent leakage (imputation/scaling within training folds only), and quantify uncertainty and calibration, especially when outputs inform clinical or translational decisions. Graph-based methods add biological structure and can improve target prioritization, but they require rigorous inductive evaluation (held-out nodes/edges) and explicit handling of network bias (e.g., degree effects and literature bias in PPIs).

Finally, interpretability should be treated as an end-to-end requirement rather than a post hoc add-on. Stable explanations typically combine model-based attributions (e.g., SHAP) with pathway enrichment and orthogonal evidence (perturbation, chemoproteomics, or independent cohorts). In practice, the most valuable AI/ML systems are those that are reproducible, auditable, and decision-aligned, rather than those that maximize a single metric under idealized splits.

From a translational perspective, the effectiveness of these tools depends on the task being addressed. For identification and quantification support, spectrum-prediction tools are often the most immediately impactful. For pathway-level interpretation in signaling studies, phosphoproteomics-focused models may provide additional mechanistic value. For target prioritization and druggability assessment, structure-based models such as AlphaFold are useful when combined with orthogonal evidence, including chemoproteomics, functional assays, and network analysis. No single model is universally optimal; performance and utility remain strongly dependent on data modality, validation design, and deployment context.

## 18. Limitations and Open Methodological Challenges in AI-Enabled Proteomics

Despite rapid methodological progress, the application of AI/ML in proteomics-driven drug discovery remains constrained by several structural and domain-specific challenges that warrant critical consideration.

**Small n versus large p regimes**

Proteomics datasets frequently exhibit high dimensionality (thousands of quantified proteins or PTM sites) relative to modest cohort sizes, particularly in translational or early-phase drug studies. This *p* ≫ n setting amplifies overfitting risk, inflates apparent performance under naïve validation, and destabilizes feature selection. While deep learning architectures are often proposed as universal solutions, empirical benchmarking suggests that under strict nested cross-validation and cohort-level holdouts, regularized linear models and gradient-boosted trees remain competitive. Model complexity does not compensate for limited statistical power.

**Correlated protein features and modular biology**

Proteins function in pathways and complexes, leading to substantial correlation structures in abundance matrices. Highly correlated features can destabilize variable importance measures, inflate confidence in individual biomarkers, and cause attribution methods (e.g., SHAP, permutation importance) to redistribute credit unpredictably across feature groups. Proteomics-specific modeling therefore requires pathway-level aggregation, stability analysis, and cautious interpretation of single-protein claims.

**MNAR missingness and censoring**

Left-censored missing values arising from detection limits are common in bottom-up MS proteomics. Unlike typical MAR assumptions in generic ML workflows, proteomic missingness is often intensity-dependent (MNAR), meaning that naïve imputation can induce artificial separability between groups. Although censor-aware methods and hurdle models exist, systematic comparison across realistic drug-discovery scenarios remains limited. Sensitivity analyses across multiple defensible preprocessing strategies are therefore essential.

**Domain shift across laboratories and acquisition regimes**

AI models trained on data from a single laboratory, instrument, or acquisition protocol may not generalize across centers. Differences in sample preparation, chromatography, fragmentation methods, collision energies, and labeling chemistries introduce distributional shifts that challenge model transportability. Spectrum prediction models, batch correction approaches, and biomarker classifiers are all susceptible to such domain shift. Robust evaluation requires site-level or time-split validation rather than random sample splits.

**Limited standardized benchmarks**

Unlike genomics and computer vision, proteomics lacks universally adopted benchmarking frameworks for downstream ML tasks such as biomarker discovery, target prioritization, or drug-response prediction. Public resources (e.g., CPTAC cohorts, synthetic peptide libraries) support evaluation of identification and spectral prediction tools, but standardized comparative benchmarks for translational ML tasks remain underdeveloped. This complicates objective comparison of algorithmic advances.

**Publication bias and over-reporting of performance gains**

Reported improvements in AI-enabled proteomics often emphasize discrimination metrics (e.g., AUC) without adequate calibration assessment, uncertainty quantification, or external validation. Positive results are more likely to be published, while failed generalization under domain shift may remain unreported. As a result, the true incremental benefit of increasingly complex architectures may be overestimated.

**Interpretability instability**

Model explanations in high-dimensional proteomics are frequently unstable across resampling, particularly when features are correlated and sample sizes are limited. Attribution consistency across folds, stability selection, and pathway-level reproducibility are rarely reported systematically. For translational applications, explanation stability should be treated as a primary evaluation criterion rather than a post hoc visualization exercise.

**Concluding perspective**

Collectively, these limitations highlight that methodological rigor, validation design, and uncertainty quantification are at least as important as algorithmic sophistication. Progress in AI-enabled proteomics will depend not only on novel architectures but also on standardized benchmarking, transparent reporting, and domain-aware evaluation frameworks that reflect the realities of drug discovery.

## 19. Where AlphaFold and DeepPhospho Fit

**AlphaFold:** Structural predictions from AlphaFold add context to proteomic differences. Mapping variants and PTM clusters onto predicted 3D structures can reveal pocket proximity, interface disruption, and allosteric pathways. Residue-level descriptors (e.g., solvent exposure, secondary structure, disorder, interface propensity) integrate seamlessly into boosted trees or GNNs for ligandability and druggability ranking. Structure-informed features further refine chemoproteomic hit triage and molecular docking decisions.

**DeepPhospho:** DeepPhospho applies deep learning to predict phosphopeptide spectral properties and site behavior, increasing identification and quantification in DDA/DIA workflows. When combined with motif- and context-based tools (e.g., NetworKIN, MusiteDeep), it strengthens kinase–substrate inference, enhancing mechanism-of-action modeling and improving pathway-level readouts in oncology and immunology applications.

**Limitations and safeguards.** For AlphaFold-derived features, confidence metrics (pLDDT and PAE) should gate downstream use; disordered regions and conformational heterogeneity can limit pocket/interface inference, and ligand-bound or PTM-dependent conformations may not be represented by a single predicted structure. For DeepPhospho and related predictors, performance can depend on training data coverage and experimental context (instrument type, collision energy, labeling/chemistry); when predictions drive biological conclusions, they should be validated against held-out experimental settings and benchmarked on the relevant acquisition regime.

In practical proteomics workflows, tools such as Prosit, DeepPhospho, and AlphaFold serve complementary rather than interchangeable roles. Prosit and related deep learning models are most effective at the spectrum level, where they improve peptide identification, fragment-ion intensity prediction, and retention time estimation. DeepPhospho is more specifically useful in phosphoproteomics, where deep models can support phosphopeptide characterization and strengthen kinase-signaling interpretation. By contrast, AlphaFold does not improve peptide identification directly; instead, it adds structural context by enabling the mapping of PTM sites, sequence variants, and candidate drug-binding regions onto predicted protein structures. Accordingly, these tools should be viewed as components of different analytical layers: spectral prediction, PTM interpretation, and structure-guided target assessment.

## 20. Emerging Perspectives and Open Challenges

The topics in this section are presented as emerging perspectives rather than mature best-practice recommendations, because systematic benchmarking in proteomics-driven drug discovery remains limited.

**Contrastive and metric learning:** Learn representations invariant to nuisance factors by pairing pre- vs. on-treatment or case–control samples [60]. Targeted proteomic assay development can also support the experimental verification of prioritized protein candidates and biomarker panels identified through AI/ML-guided analyses [61].

**Causal dynamics:** Dynamic Bayesian networks and ODE-informed neural models reconstruct signaling flows from time-course proteomics.

**Diffusion models for spectra:** Generative diffusion approaches improve denoising and augmentation for low-abundance peptides.

**Topological data analysis:** Mapper and persistent homology detect rare cellular states such as emergent drug resistance.

**Federated learning and privacy:** Federated training enables cross-institutional modeling without centralizing data, supporting multi-center trials and safeguarding privacy.

Standardized benchmarking across labs and acquisition modes (DDA/DIA), transparent reporting of preprocessing and validation protocols, and evaluation under domain shift remain open challenges. Progress will likely depend on shared reference datasets, stronger uncertainty quantification, and privacy-preserving multi-center learning that supports clinically realistic validation without centralizing sensitive data.

## 21. Practical Checklist (for Teams)

- **Start with strong baselines:** Establish elastic-net and XGBoost models under strictly nested cross-validation.
- **Leverage topology:** Add network propagation methods and GNNs when biological graph structure (e.g., PPIs, kinase networks) is relevant.
- **Exploit deep priors:** Use Prosit or DeepPhospho for spectra and PTMs, protein language models for sequence context, and AlphaFold for structural features.
- **Control noise:** Apply censor-aware imputation, batch correction, and mixed-effects models where study design requires it.
- **Explain and quantify:** Employ SHAP, pathway attribution, and conformal prediction intervals to provide transparent, case-level explanations and confidence.
- **Design for translation:** Favor parsimonious biomarker panels, assess assay feasibility, and test generalization with leave-site-out validation.
- **Govern and reproduce:** Track data and models, containerize environments, and implement workflows with Nextflow or Snakemake to ensure reproducibility and auditability.

## 22. Tables and Boxes

Table 1 provides a decision-oriented comparison of the major AI/ML model families reviewed in this manuscript. For each family, we summarize the typical data and cohort setting in which the method is most appropriate, its principal strengths and limitations, the recommended validation approach, the best use case, and the drug-discovery decision it most directly supports. Researchers can use this table as a practical reference for method selection before undertaking a new analytical project. The table should be read alongside Section 23, which discusses comparative performance evidence across tasks.

Taken together, Table 1 illustrates that no single model is universally optimal for proteomics-driven drug discovery. Instead, method selection should be driven by cohort size, feature dimensionality, interpretability requirements, and the downstream decision the model is intended to support. For most tabular proteomics tasks with moderate sample sizes, starting with elastic-net regression and gradient-boosted trees as mandatory baselines before considering more complex architectures is strongly recommended.

Table 2 provides a comparative overview of key proteomics software tools and platforms, organized by analytical task. The table is intended to help researchers identify which tools best match their experimental workflow and computational requirements. Tools differ substantially in their primary strengths and limitations, and researchers should consider their acquisition strategy, cohort scale, and downstream analysis goals when selecting platforms.

As shown in Table 2, no single tool spans the full analytical workflow from raw spectra to translational decision. In practice, effective proteomics-to-drug-discovery pipelines require a combination of tools across data processing, quantification, targeted assay validation, and ML governance layers. The workflow and MLOps tools listed in the final row (Nextflow, Snakemake, Docker, MLflow, DVC) are critical for ensuring that multi-tool analyses remain reproducible and auditable.

## 23. Comparative Performance of AI/ML Methods in Proteomics

Across proteomics-driven drug discovery tasks, the relative performance of AI/ML methods depends strongly on data modality and cohort size. For tabular proteomics datasets, such as protein or PTM abundance matrices, regularized linear models and gradient-boosted trees often provide strong and competitive baselines, particularly when the number of quantified proteins or PTM sites exceeds the number of samples. Reported performance varies substantially across cohorts, endpoints, preprocessing workflows, and validation designs; therefore, discrimination metrics such as AUC should be interpreted together with calibration, feature stability, and external or site-level validation [6,7,8,13].

In contrast, deep learning models show clear advantages in spectrum-level tasks, such as peptide identification and retention time prediction, where large-scale training data are available. For example, Prosit has been reported to substantially improve peptide identification rates and fragment ion intensity prediction accuracy in ProteomeTools benchmark datasets, with reported gains varying by dataset, acquisition regime, and evaluation criteria [46]. Cross-laboratory generalization of these gains remains variable and depends on instrument type and acquisition settings.

Graph neural networks provide additional value when biological network structure is incorporated, supporting target prioritization and drug–target interaction prediction. However, their performance is sensitive to network quality and evaluation design, and gains are often context-dependent [32].

Overall, these findings highlight that no single model dominates across all tasks, and that model selection should be guided by data characteristics, cohort size, and the specific biological question.

In practical terms, regularized linear models and gradient-boosted trees are often the most useful first-line methods for tabular protein- or PTM-abundance matrices, especially when sample size is modest and interpretability is required. Deep learning is more appropriate when large-scale spectra, imaging data, sequence information, or transfer-learning resources are available. Graph neural networks are most useful when high-quality biological networks or drug–target–disease graphs are available and when the goal is target ranking or repurposing rather than simple classification. Causal inference methods are most valuable when the central question is whether a protein is likely to be a driver rather than a correlate. Thus, model choice should be determined by data modality, cohort size, validation design, and the downstream drug-discovery decision.

Before these approaches can be considered ready for broader translational use, proteomics-based ML studies should align with emerging reporting and risk-of-bias standards for AI-enabled prediction models. Recent guidance such as TRIPOD+AI emphasizes transparent reporting of model development, validation, preprocessing, missing-data handling, and performance evaluation [62], while PROBAST+AI provides a framework for assessing risk of bias and applicability in prediction-model studies [63]. In proteomics, these general AI-reporting principles should be combined with domain-specific requirements, including peptide/protein FDR control, quantification uncertainty, batch-aware validation, and external testing using benchmark resources such as CPTAC [64]. Together, these practices can improve reproducibility, reduce overclaiming, and make comparisons between proteomics ML methods more reliable.

## 24. Conclusions

AI/ML methods are becoming increasingly important components of proteomics-driven drug discovery. Classical linear and kernel models provide transparent and robust baselines; ensemble methods capture nonlinear interactions; dimensionality reduction and clustering reveal biological programs and subtypes; deep learning is particularly valuable for spectra, images, sequences, and PTM-focused tasks; graph methods integrate biological topology; and causal and survival frameworks align analysis with translational decision-making. Tools such as DIA-NN, FragPipe, Skyline, MSstats, Prosit, DeepPhospho, and AlphaFold support different layers of the proteomics workflow, from raw spectra to target hypotheses, biomarker panels, and repurposing candidates. However, successful translation depends less on algorithmic complexity alone than on rigorous validation, censor-aware preprocessing, domain-shift-aware benchmarking, calibration, interpretability, reproducibility, and experimental confirmation. Future progress will require standardized benchmarks, stronger uncertainty quantification, and decision-oriented workflows that connect ML predictions to experimentally testable drug-discovery actions.

## Figures and Tables

**Table 1:** Decision-oriented comparison of AI/ML model families for proteomics-driven drug discovery.

Model Family	Representative Algorithms	Typical Data/Cohort Setting	Strengths	Key Limitations	Recommended Validation	Best Use Case	Drug-Discovery Decision Supported
Linear baselines	Logistic regression, lasso, ridge, elastic-net, elastic-net Cox	Small to moderate cohorts; high-dimensional protein or PTM matrices	Interpretable, stable in *p* ≫ *n* settings, useful for sparse feature selection	Limited nonlinear modeling; sensitive to preprocessing and correlated features	Nested cross-validation; site, batch, or time holdout; coefficient stability analysis	Biomarker panels, transparent risk scores, survival models	Select proteins for targeted assay validation; support interpretable biomarker development
Support vector machines	Linear SVM, RBF-SVM	Small to moderate cohorts with many features	Strong classification performance in high-dimensional data; effective for margin-based separation	Requires scaling and imputation; kernel models can be difficult to interpret and tune	Nested cross-validation; batch/site holdout; sensitivity to scaling and missingness	Disease classification, mechanism-of-action signatures	Classify disease or treatment-response groups; support mechanism-of-action stratification
Tree ensembles	Random forest, ExtraTrees	Moderate-sized tabular proteomics datasets	Captures nonlinear effects and feature interactions; useful for screening candidate markers	Variable importance can be biased by correlated proteins; may overfit imputation artifacts	External or site/time holdout; permutation importance; feature-stability analysis	Feature screening, biomarker discovery, target prioritization	Prioritize candidate proteins or pathways for follow-up validation
Gradient-boosted trees	XGBoost, LightGBM, CatBoost	Moderate to large tabular datasets integrating proteomics and metadata	Strong performance on structured data; handles nonlinearities and interactions	Requires careful tuning, early stopping, and calibration; may be sensitive to batch effects	Nested cross-validation; external validation; calibration curves and Brier score	Response prediction, toxicity prediction, target ranking	Rank patients, targets, or compounds for experimental or translational prioritization
Dimensionality reduction and representation learning	PCA, ICA, NMF, autoencoders, VAEs	Moderate to large datasets; exploratory analysis or denoising	Reveals latent structure, biological programs, and technical variation	Components may mix biology and batch effects; 2D embeddings can be misused in prediction	Batch-aware evaluation; replicate consistency; avoid leakage from embeddings	Program discovery, denoising, batch assessment	Identify biological programs, disease subtypes, or quality-control problems
Clustering	k-means, k-medoids, hierarchical clustering, GMM, DBSCAN/HDBSCAN, spectral clustering	Exploratory subtype or module discovery	Useful for discovering subgroups, co-regulated modules, or tissue niches	Sensitive to missingness, scaling, distance metric, and batch effects	Batch-stratified QC; cluster stability analysis; biological replication	Subtype discovery, niche detection, protein-module discovery	Define candidate patient subgroups or biological states for downstream testing
Deep learning	CNNs, TCN/LSTM/GRU, transformers, protein language models, autoencoders	Large labeled datasets, raw spectra, imaging data, sequence/PTM tasks, or transfer-learning settings	Strong for spectra, images, sequences, and complex representations	Data hungry; lower interpretability; high domain-shift risk across instruments and laboratories	Strict instrument/site/time holdout; external benchmarks; comparison with simple baselines	Spectrum prediction, imaging proteomics, PTM modeling, sequence-informed prediction	Improve peptide identification, PTM interpretation, spatial analysis, or sequence-informed target assessment
Graph learning	GCN, GraphSAGE, GAT, R-GCN, knowledge-graph embeddings	Proteomics integrated with PPI, kinase–substrate, drug–target, or disease networks	Incorporates biological topology and relational context	Sensitive to network quality, degree bias, and literature bias; harder to validate	Inductive node/edge holdouts; degree-aware evaluation; uncertainty reporting	Target ranking, pathway inference, drug repurposing	Prioritize therapeutic targets, pathways, or drug–disease links
Calibration and uncertainty methods	Platt scaling, isotonic regression, conformal prediction, Bayesian neural networks, MC dropout, deep ensembles	Any predictive setting where probabilities guide decisions	Improves reliability of model confidence; supports decision-making under uncertainty	Requires sufficient validation data; uncertainty may fail under severe domain shift	Reliability curves, Brier score, calibration-in-the-large, decision-curve analysis	Clinical or translational prediction models	Decide whether a prediction is confident enough for experimental or clinical follow-up
Causal inference	Propensity weighting, causal forests, double/debiased ML, Bayesian networks, target-trial emulation	Observational or perturbational proteomics with confounding risk	Helps distinguish associations from plausible drivers	Requires explicit assumptions, covariate control, and sensitivity analysis	Target-trial-style design; negative controls; sensitivity analysis	Driver identification, treatment-effect estimation, confounding control	Prioritize proteins more likely to be causal drivers rather than passive correlates

**Table 2:** Comparative overview of selected proteomics tools and platforms.

Task	Representative Tools	Main Strength	Key Limitation	Best Suited for
DDA processing	MaxQuant, FragPipe	Mature protein identification and quantification	Parameter choices affect reproducibility	Discovery proteomics
DIA processing	DIA-NN, Spectronaut, OpenSWATH	High-throughput DIA quantification	Sensitive to acquisition strategy and spectral-library design	Large-scale DIA cohorts
Targeted assays	Skyline, MSstats	Assay development and statistical validation	Requires targeted validation data	Biomarker verification
Spectrum prediction	Prosit	Improves spectral library generation and peptide identification	Domain shift across instruments and acquisition settings	DIA library prediction
PTM/phosphoproteomics	DeepPhospho, PTM-Shepherd, NetworKIN	PTM characterization and kinase-signaling interpretation	Context and instrument dependence	Phosphoproteomic mechanism studies
Structure context	AlphaFold, fpocket	Maps PTMs/variants to structural features	Limited for disorder and ligand-induced conformations	Druggability assessment
Network analysis	Cytoscape, STRING, Reactome, Neo4j	Biological context and graph modeling	Network bias and incomplete coverage	Target ranking and repurposing
Workflow/MLOps	Nextflow, Snakemake, Docker, MLflow, DVC	Reproducibility and auditability	Requires computational infrastructure	Regulated or multi-site workflows

## Data Availability

No new data were created or analyzed in this study. Data sharing is not applicable to this article.

## Abbreviations

AI, artificial intelligence; ML, machine learning; DL, deep learning; MS, mass spectrometry; DDA, data-dependent acquisition; DIA, data-independent acquisition; PTM, post-translational modification; PPI, protein–protein interaction; GNN, graph neural network; PCA, principal component analysis; NMF, non-negative matrix factorization; VAE, variational autoencoder; TCN, temporal convolutional network; SRM, selected reaction monitoring; PRM, parallel reaction monitoring; FDR, false discovery rate; MAR, missing at random; MNAR, missing not at random; QC, quality control.
